# Large-diameter compression arteries as a possible facilitating factor for trigeminal neuralgia: analysis of axial and radial diffusivity

**DOI:** 10.1007/s00701-015-2673-4

**Published:** 2016-01-06

**Authors:** Wei Lin, Wan-ping Zhu, Yi-li Chen, Guo-can Han, Yue Rong, Yu-rong Zhou, Qiao-wei Zhang

**Affiliations:** Department of Radiology, Sir Run Run Shaw Hospital, School of Medicine, Zhejiang University, 3 Qingchun East Road, Hangzhou, 310016 China; Operating Room, Sir Run Run Shaw Hospital, School of Medicine, Zhejiang University, Hangzhou, China; Department of Neurosurgery, Sir Run Run Shaw Hospital, School of Medicine, Zhejiang University, Hangzhou, China

**Keywords:** Axial diffusivity, Demyelination, Neurovascular compression, Radial diffusivity, Trigeminal neuralgia

## Abstract

**Background:**

Neurovascular compression (NVC) of the trigeminal nerve is associated with trigeminal neuralgia (TN). Some arteries that compress the trigeminal nerve are large, while others are small. This study evaluated the influence of diameter of compression arteries (DCA) on NVC with and without TN using axial diffusivity (AD) and radial diffusivity (RD) of magnetic resonance (MR) imaging.

**Methods:**

Fifty TN patients with unilateral NVC, 50 asymptomatic patients with unilateral NVC, and 50 healthy controls (HC) were divided into three groups (NVC with TN, NVC without TN, and HC). The three groups were imaged with a 3.0-T MR system using three-dimensional fast imaging employing steady-state acquisition (3D FIESTA) and diffusion tensor imaging (DTI). We compared the mean size of DCA between NVC with and without TN. The mean values of AD and RD at the site of NVC were compared between the three groups. Correlation analyses were performed between the DCA and the diffusion metrics (AD and RD) in NVC patients with and without TN.

**Results:**

The mean DCA in NVC patients with TN (1.58 ± 0.34 mm) was larger than that without TN (0.89 ± 0.29 mm). Compared with NVC without TN and HC, the mean values of RD at the site of NVC with TN were significantly increased; however, no significant changes of AD were found between the groups. Correlation analysis showed that DCA positively correlated with RD in NVC patients with and without TN (*r* = 0.830, *p* = 0.000). No significant correlation was found between DCA and AD (*r* = 0.178, *p* = 0.077).

**Conclusions:**

Larger-diameter compression arteries may increase the chances of TN, and may be a possible facilitating factor for TN.

## Introduction

Neurovascular compression (NVC) as a cause of trigeminal neuralgia (TN) is a widely accepted mechanism [3, 6]; however, amongst all the offending arteries, some are big, while others are small. Is the size of the arteries different between NVC with TN and NVC without TN? To our knowledge, there is no study that answers this particular question. In this study, we aimed to evaluate diameter of compression arteries (DCA) between NVC with TN and without TN, and to investigate whether the size of the DCA has any influence on TN, we compared diffusion metrics (axial diffusivity [AD] and radial diffusivity [RD]) between three groups (NVC with TN, NVC without TN, and healthy control [HC] subjects). Further, correlation analyses were performed between the DCA and diffusion metrics.

## Materials and methods

### Participants

The study was conducted between February 2012 and June 2015. We recruited 50 TN patients with unilateral NVC (19 men, 31 women; age range, 52–82 years, mean age, 57.98 ± 5.46 years). The diagnosis of all primary TN patients was confirmed in accordance with the diagnostic criteria of typical TN [15] and treated with microvascular decompression (MVD) at the Department of Neurosurgery, Sir Run Run Shaw Hospital. To validate the study, 50 patients with unilateral NVC without TN (20 men, 30 women; age range, 50–80 years, mean age, 57.08 ± 4.76 years) and 50 HC subjects (21 men, 29 women; age range, 50–66 years, mean age, 56.88 ± 3.99 years) were also included in the study. Both of these groups came from the health care center of our hospital and underwent magnetic resonance imaging (MRI) for a routine health check-up. Patients with different illnesses, such as arteriovenous malformations, bony anomalies, hemorrhage, ischemia, multiple sclerosis lesions, cysts, any type of intracranial mass, head trauma, head operation, other disorders that might affect central nervous system function, presence of metal implants, and patient motion during examinations, were excluded. Only healthy controls showing no signs of neurovascular conflict were included. Low-quality images or images containing artifacts were also excluded. Written informed consent was obtained from each participant before entering the study, and the study was approved by the institutional ethics committee.

### Magnetic resonance imaging

All participants were imaged with a 3.0-T magnetic resonance scanner (Signa Excite HDx 3.0, GE Medical Systems, Milwaukee, WI, USA) using three-dimensional fast imaging employing steady-state acquisition (3D FIESTA) and diffusion tensor imaging (DTI). An eight-channel head coil was used with foam padding and braces to restrict head motion. The following imaging protocols were used: (a) 3D FIESTA sequence with repetition time/echo time (TR/TE) = 6.1 ms/1.5 ms, flip angle = 60°, field of view = 240 mm × 240 mm, matrix = 512 × 512, and two acquisitions; (b) DTI with a single-shot spin echo echo-planar imaging protocol, TR/TE = 10,000 ms/96.6 ms, field of view = 240 mm × 240 mm, matrix = 128 × 128, *b* = 0 and 1000 s/mm^2^ with diffusion gradients applied in 20 non-collinear directions, 19 slices of 2.4 mm without gap, and acquisition time = 5.12 min.

### DTI data processing

**Fig. 1:**
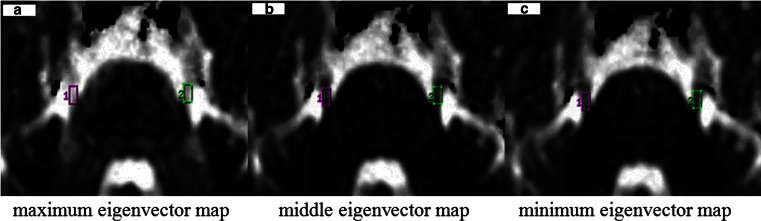
We placed a box-shaped ROI over the root entry zone of the trigeminal nerves to measure λ1, λ2, and λ3 in the maximum, middle, and minimum eigenvector maps, respectively. The ROI size of the *left* and *right* side was equivalent (approximately 30 mm^2^)

The trigeminal nerve was easily delineated in all participants by applying all MRI sequences. The diagnostic criteria [14] of NVC were defined as no visible cerebrospinal fluid between the trigeminal root and its adjacent arteries on 3D FIESTA MRI (Fig. 4b1, c1). The mean sizes of DCA were measured at the site of NVC by 3D FIESTA. The original DTI data were processed with Functool software in an AW4.4 workstation (GE Medical Systems, Milwaukee, WI, USA) to generate fractional anisotropy (FA) (Fig. 4a2, b2, c2) maps, maximum (λ1), middle (λ2), and minimum (λ3) eigenvector maps (Fig. 1). We placed box-shaped regions of interest (ROIs) over the root entry zone of the trigeminal nerve to measure λ1, λ2, and λ3 (Fig. 1). For each ROI (approximately 30 mm^2^), the AD and RD were calculated based on the following equations [1, 2, 16–19]: AD = λ1, RD = (λ2 + λ3)/2

### Statistical analysis

All statistical calculations were performed with SPSS 16 software (SPSS Inc., Chicago, IL, USA). The DCA and all diffusion metrics, including AD and RD were measured and calculated independently by two observers who were blinded to the clinical features and purpose of this study. For statistical analysis, we utilized the mean values from the two observers. Table 1 compares the mean of DCA between two groups (NVC with TN and without TN); AD and RD are compared between the three groups (NVC with TN, NVC without TN, and HC) using a one-way analysis of variance (ANOVA) followed by Bonferroni’s multiple comparison tests. Results are expressed as mean ± SD (Table 1). *p* values <0.05 were considered statistically significant. The correlation analysis between DCA and diffusion metrics (AD and RD) was ere also performed.

**Table 1:** Mean values of DCA, AD, and RD in the three groups (NVC with TN, NVC without TN, and HC)

Variable	NVC with TN	NVC without TN	HC	NVC with TN vs. NVC without TN	NVC with TN vs. HC	NVC without TN vs. HC
DCA(mm)	1.58 ± 0.34	0.89 ± 0.29	–	*p* = 0.00	–	–
RD (×10^−3^mm^2^/s)	1.69 ± 0.21	1.17 ± 0.13	1.15 ± 0.24	*p* = 0.00	*p* = 0.00	*p* = 0.34
AD (×10^−3^mm^2^/s)	2.91 ± 0.10	2.91 ± 0.11	2.89 ± 0.07	*p* = 0.78	*p* = 0.16	*p* = 0.27

*DCA* diameter of compression arteries, *AD* axial diffusivity, *RD* radial diffusivity, *NVC* neurovascular compression, *TN* trigeminal neuralgia, *HC* healthy controls

*p* < 0.05 was considered significant

## Results

### Offending arteries and compression site of NVC

Based on the 3D FIESTA MRI findings, the trigeminal nerve could be easily delineated on both sides in all the three groups (NVC with TN, NVC without TN, and HC). All HC did not present with NVC. In more than half of the NVC patients with and without TN (82/100, 82 %), contact with the superior cerebellar artery (SCA) was identified, other patients had nerve contact with other arteries including the anterior inferior cerebellar artery (AICA) (14/100, 14 %), vertebral artery (VA) (2/100, 2 %), and posterior inferior cerebellar artery (PICA) (2/100, 2 %). In most of the NVC patients with TN, the proximal part of the arteries caused compression (49/50, 98 %), and only in one patient the distal part of VA caused compression (1/50, 2 %); while in NVC patients without TN, compression was induced by the distal part of the arteries (40/50 patients, 80 %), proximal part of the arteries (3/50, 6 %), and artery branch (7/50, 14 %).

### DCA and diffusion metrics

Table 1 summarizes the DCA and diffusion metrics of our study. The mean DCA (1.58 ± 0.34 mm) was larger in NVC patients with TN than those NVC patients without TN (0.89 ± 0.29 mm). Compared with the NVC without TN (RD = 1.17 ± 0.13) and HC (RD = 1.15 ± 0.24), the mean values of RD (1.69 ± 0.21) at the site of the NVC with TN were significantly increased; however, no significant changes of AD was found between the three groups (AD = 2.91 ± 0.10, 2.91 ± 0.11, 2.89 ± 0.07, respectively).

### Correlation between DCA and diffusion metrics

**Fig. 2:**
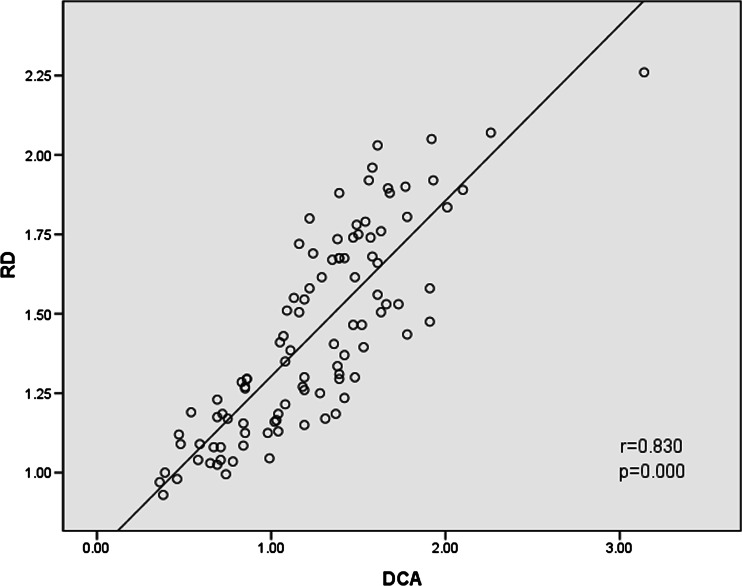
Correlation analyses between RD and DCA in NVC patients with and without TN (*n* = 100). Note: *RD* radial diffusivity (×10^−3^mm^2^/s), *DCA* diameter of compression arteries (mm), *NVC* neurovascular compression, *TN* trigeminal neuralgia

**Fig. 3:**
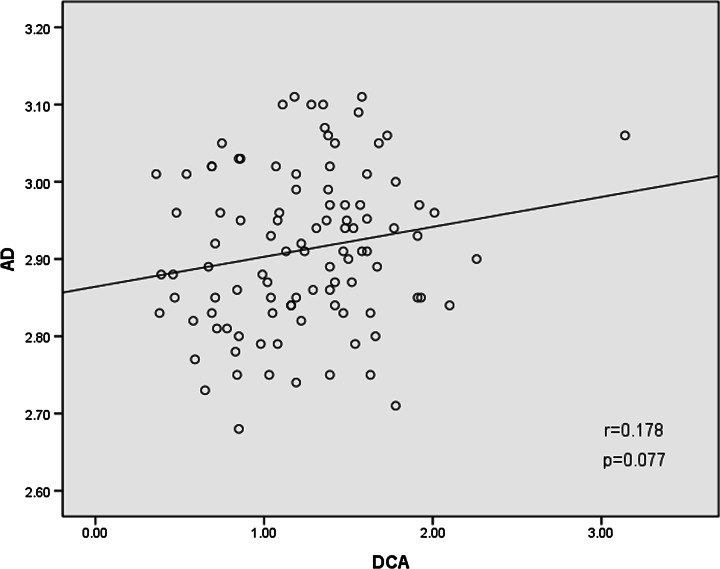
Correlation analyses between AD and DCA in NVC patients with and without TN (*n* = 100). Note: *AD* axial diffusivity (×10^−3^mm^2^/s), *DCA* diameter of compression arteries (mm), *NVC* neurovascular compression, *TN* trigeminal neuralgia

The correlation analysis showed that DCA significantly and positively correlated with RD in NVC patients with and without TN (RD and DCA: *r* = 0.830, *p* = 0.000; Fig. 2). The correlation between DCA and AD were also calculated, which showed no significant correlation (AD and DCA: *r* = 0.178, *p* = 0.077, Fig. 3).

## Discussion

### Compression site and DCA in NVC with and without TN

TN is an unbearable pain syndrome caused by several mechanisms. Compression of trigeminal nerves by the arteries is considered one of the primary causes [3, 6]. TN is also considered to be related to the size of DCA. The aim of this study was to assess the mean sizes of DCA between NVC with and without TN, and to investigate microstructural tissue changes of trigeminal nerve using AD and RD of MRI. To further explore the relationship between the DCA and TN, correlation analyses were performed between DCA and diffusion metrics (AD and RD).

In our study, compression by the proximal part of the arteries was observed in most of the NVC patients with TN (49/50, 98 %), while compression by the distal part of the arteries was observed in most of the NVC patients without TN (40/50, 80 %). 3D FIESTA can show the diameter of compression arteries clearly, the mean DCA with TN was 1.58 ± 0.34 mm, and mean DCA without TN was 0.89 ± 0.29 mm. The DCA with TN was significantly larger than that without TN (Table 1). The large DCA may have an effect on the occurrence of TN.

### Diffusion metrics in NVC with and without TN

We showed that AD and RD of DTI are useful in detecting microstructural changes of trigeminal nerve in the three groups. A study by Liu et al. [12] has suggested that specific diffusion metrics of AD and RD may be used to specifically assess trigeminal nerve change. AD = λ1, where λ1 represents the water diffusivity parallel to the axonal fibers, and therefore reflects axonal changes of the cerebral white matter (WM). RD = (λ2+λ3)/2, where λ2 and λ3 represent water diffusion in the planes orthogonal to the long axis of the axon, which thus reflects myelin changes (such as demyelination and remyelination) of cerebral WM, and eigenvalues (λ1, λ2, and λ3) are the magnitudes of eigenvectors [1, 17, 19].

Table 1 summarizes the detailed results of our study. Compared with NVC without TN (RD = 1.17 ± 0.13) and HC (RD = 1.15 ± 0.24), the mean values of RD (1.69 ± 0.21) at the site of the NVC with TN were significantly increased; however, no significant change of AD was found between the three groups (AD = 2.91 ± 0.10, 2.91 ± 0.11, 2.89 ± 0.07, respectively). Increased RD indicates that there is focal demyelination at the compressed sides of NVC with TN. Compared with HC, no significant difference in RD means there is no focal demyelination at the compressed sides of NVC without TN.

### Correlation between DCA and diffusion metrics

Liu et al. [12] reported that, compared with HC and the uncompressed side, the compressed side showed significantly increased RD and unchanged AD. Their observations suggested that the primary TN pathology is the focal demyelination of the sensory axons at the site of the NVC, and they further indicated that demyelination without significant axonal injury is an essential pathological basis of the compressed trigeminal nerve. AD and RD have been used to characterize trigeminal nerve damage from NVC. Our correlation analysis showed that DCA significantly and positively correlated with RD in NVC patients with and without TN (RD and DCA: *r* = 0.830, *p* = 0.000; Fig. 2). Large arteries are more likely to cause higher RD and more easily to make trigeminal nerve demyelination, while small arteries are not. Some studies [4, 5, 10, 12] showed that trigeminal nerve demyelination is prevalent in TN patients. Demyelination causes destruction of the trigeminal nerve integrity, which leads to TN. Hence TN may be the result of chronic and long-term physical compression of the large diameter of the arteries. The correlation between DCA and AD were also calculated, which showed no significant correlation (AD and DCA: *r* = 0.178, *p* = 0.077; Fig. 3), NVC might rarely cause trigeminal axon damage.

### Possible causes of TN with larger DCA

**Fig. 4:**
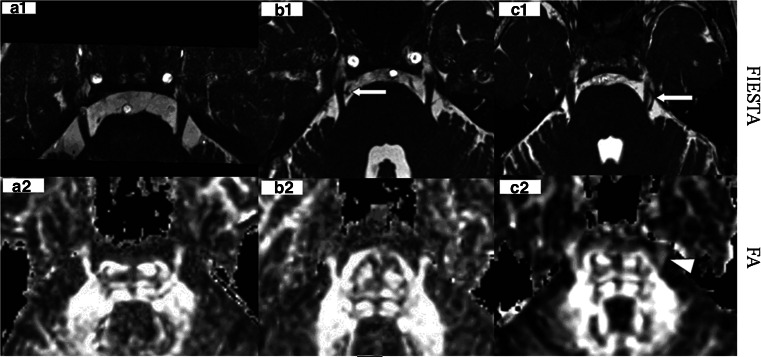
**a1** 3D FIESTA MR image showing a 53-year-old man (HC without NVC). **a2** fractional anisotropy image showing same signal in both *left* and *right* trigeminal nerve; **b1** an artery branch (*arrow*) (DCA: 0.70 mm) compressing the *right* trigeminal nerve root in a 52-year-old asymptomatic woman (NVC without TN). **b2** fractional anisotropy image showing same signal in both *left* and *right* trigeminal nerves; **c1** the proximal SCA (*arrow*) (DCA: 1.56 mm) compressing the *left* trigeminal nerve root in a 58-year-old TN woman (NVC with TN). **c2** fractional anisotropy image showing hypointensity signal in the *left* nerve (*arrowhead*) compared with the opposite side. Note: *HC* healthy controls, *NVC* neurovascular compression, *DCA* diameter of compression arteries, *TN* trigeminal neuralgia, *SCA* superior cerebellar artery

According to Kamiguchi et al. [7], NVC is caused by elongation, tortuosity, dilatation, or variant arteries. Regarding vascular compression of the trigeminal nerve, some patients may have symptoms, while others are asymptomatic [5, 11, 13]. Why? There are many reasons for answering these questions. Some studies reported [4, 5, 10] that TN is likely a result of chronic vascular compression. Other study suggested that symptoms may be related to the degree of vascular compression, the more serious the trigeminal nerve is depressed, the greater is the presence of clinical symptoms [3]. However, no study has shown whether the size of DCA has some influence on TN. Miller et al. [13] reported patients with TN with NVC that is, on average, more proximal to the nerve than patients without facial pain. Our study showed that the NVC of TN patients was often from the proximal part of the arteries. The DCA of proximal arteries is bigger than the distal ones. The size of DCA at the site of NVC may influence the severity of a neurovascular conflict [8]. Leal PR et al. [9] suggested that atrophic changes in trigeminal nerves, which significantly correlated with the severity of the NVC. Our study showed that DCA significantly and positively correlated with RD in NVC patients with and without TN (Fig. 2). Increased RD indicates that there is focal demyelination at the compressed sides of NVC with TN [12]. Due to its incessant pulsations, the large DCA (Fig. 4c1), such as the proximal part of the offending arteries, may have sufficient and chronic physical pressure on the trigeminal root that causes chronic injury to the myelin sheath and leads to trigeminal nerve focal demyelination and loss of anisotropy. In our study, the fractional anisotropy image shows hypointensity signal in the compressed nerve compared with the opposite side (Fig. 4c2), and ultimately leads to TN, while smaller DCA such as the distal part of the offending arteries and artery branch (Fig. 4b1) may therefore have insufficient pressure on the trigeminal nerve, which may not cause focal demyelination and in turn does not lead to TN; there is no signal intensity change in fractional anisotropy image (Fig. 4b2). According to our study results, the larger the DCA in the NVC, the more are the chances of suffering from TN. The larger DCA might play an important role in TN.

## Conclusions

The mean DCA in NVC patients with TN were larger than those without TN. The mean values of RD at the site of the NVC with TN were significantly increased than both NVC without TN and HC. However, no significant changes of AD were found between the three groups. Our study also showed that DCA significantly and positively correlated with RD in NVC patients with and without TN. Our study showed that larger DCA may increase the chance of trigeminal nerve demyelination, and may be a possible facilitating factor for TN.
